# Comparison of Sublimation 3D Scanning Sprays in Terms of Their Effect on the Resulting 3D Scan, Thickness, and Sublimation Time

**DOI:** 10.3390/ma16186165

**Published:** 2023-09-11

**Authors:** Jakub Franke, Tomáš Koutecký, Daniel Koutný

**Affiliations:** Institute of Machine and Industrial Design, Faculty of Mechanical Engineering, Brno University of Technology, Technická 2896/2, 616 69 Brno, Czech Republic; jakub.franke@vut.cz (J.F.); daniel.koutny@vut.cz (D.K.)

**Keywords:** optical 3D scanning, matte coating, scanning spray, sublimation coating, coating thickness

## Abstract

This study compared eight sublimation scanning sprays in terms of their effect on 3D scanning results, coating thickness, and sublimation time. The work used an automated spraying system to ensure the same deposition conditions for all tested materials. All experiments were performed under the same environmental conditions to exclude the influence of the ambient environment on the coatings. All tested scanning sprays created coatings with thicknesses in the order of tens of micrometers that were detectable by the 3D scanner Atos III Triple Scan. The coatings must be applied carefully when accurate measurements are required. All used materials enabled the capture of the highly reflective surface of the Si-wafer. However, the differences between some sprays were significant. Sublimation time measurements showed that all coatings disappeared from the Si-wafer surface completely. Nevertheless, all coatings left visible traces on the mirror-like surface. They were easily wiped off with a cloth.

## 1. Introduction

Optical 3D scanners are used in a wide range of applications for their ability to digitize physical objects into the virtual environment. They are typically used for reverse engineering, geometric and dimensional inspection, medical purposes, etc. This work focused on active optical triangulation-based 3D scanners, specifically structured light scanners. The working principle of these scanners is described in the work of Sansoni [1]. The scanner consists of a projector and camera/s. The projector projects a light pattern on the scanned object, which is deformed by the object geometry, and the cameras capture the scene. The orientation and position of the projector and cameras in the scanner body are known thanks to the calibration. It enables the reconstruction of the scanned objects by triangulation.

The advantages of structured light scanners are their scanning speed and the fact that they are non-contact. However, they have limitations when objects with problematic surfaces must be scanned, typically specular (reflective), translucent, transparent, or dark surfaces [2]. It is coupled with the working principle of these scanners when the projected light is reflected from the scanned object. A proper reflection from the scanned surface is thus essential. Phenomena coupled with the scanning of the specular surface were described in the work of Wang and Feng [3]. In this case, the most problematic is a specular reflection. In the work of Chen [4], the effect of the translucent surface on the results of structured light 3D scanning was discussed. Projected light can penetrate the translucent surface, resulting in the wrong computation of a depth coordinate of the scanned point cloud, which can lead to a non-accurate 3D scan. In the case of transparent surfaces, conventional scanners fail completely. The problem with dark surfaces is that they absorb the projected light and thus reduce the amount of reflected light into the cameras of the scanner. The influence of the scanned surface color on the results of 3D scanning was investigated in the work of Zaimović-Uzunović and Lemeš [5]. All problematic surfaces generally lead to 3D scans with errors or scans with insufficient information about the digitized surface.

There are two approaches for digitizing problematic objects using the 3D scanner. The first one is based on using special 3D scanning techniques. The main drawback of this approach is that each type of problematic surface requires a different scanning technique with specific hardware and software. This is reported in the work of Ihrke [6]. The second approach is based on using a temporary matte coating. In this case, the scanned surface is covered by a matte coating, which improves its optical properties and enables its digitization. The main advantage of this approach is that it is usable for all conventional 3D scanners. The disadvantage of this approach is that the applied coating can change the geometry of the scanned object because it is an added material on the surface of the scanned body, as was stated in the work of Rukosuyev [7]. For accurate 3D scanning, the coating thickness must be as thin as possible and at the same time must sufficiently improve the optical properties of the scanned surface. The topic of the fine even coating was also the scope of the work of Maeng and Lee [8]. They explored the usability of electrostatic powder coating for 3D scanning purposes. In this study, both coating and removal process were investigated.

The work of Paloušek [9] was focused on the effect of matte coating on the accuracy of optical 3D scanning. A chalk spray and titanium dioxide (TiO_2_) deposited by an airbrush gun were used as the coating materials. The coating thickness of the chalk spray was around 44 µm, while the titanium-based coating was almost ten times thinner. The coating thickness values were obtained based on the 3D scanning of the coated gauges. Another paper dealing with TiO_2_-based coatings and their effect on 3D scanning accuracy is the work of Franke [10]. This work was focused on optimizing the process of matte coating deposition in terms of coating thickness and matting ability. The resulting coatings had a thickness below 1 µm and provided sufficient optical properties for accurate 3D scanning. The coating thickness in this study was measured by stylus profilometer. The work of Pereira [2] is another study focusing on the effect of the coating material on 3D scanning accuracy. The gold, silver, platinum, and carbon deposited by sputtering were used as coating materials. All these materials were suitable for accurate 3D scanning and measurements with a tolerance of up to 0.01 mm, compared to the traditionally used developer. The effect of the matte coating on the 3D scanning accuracy in dental applications was discussed in the work of Burde [11]. Aerosol sprays, such as Helling 3D Laser Scanning Anti-Glare and Digiscan-Spray, were used as matting agents. Both sprays had acceptable coating thickness for clinical use. The coating thickness was around 16.1 µm for the Helling spray and 13.6 µm for the Digiscan spray. The coating thickness values were obtained by comparing the 3D scans of the non-coated and coated part. The work of Yang [12] studied the effect of the chalk spray coating on the 3D scanning results. In this case, the effects of spray condition and spray operator skills on the precision of 3D scanning were investigated.

From the nature of the previously mentioned coating materials, there is one more disadvantage of matte coatings. Cleaning must be performed to remove the coating from the surface of the scanned object. The cleaning is generally conducted by wiping with a cloth or with a brush or by washing. Sometimes, an ultrasonic cleaner must be used. However, in some cases, even an ultrasonic cleaner cannot completely remove the coating material from the surface. This is typical for TiO_2_-based coatings and their application on rubber surfaces, rough surfaces, surfaces with complex geometry, etc. This disadvantage can be eliminated by using sublimation scanning sprays, which spontaneously vanish from the scanned surface after 3D scanning. Sublimation coatings were investigated in the work of Díaz-Marín [13]. In this case, cyclododecane was used as a coating agent for the 3D scanning of archaeological objects. The sublimation rate measurement in this study was based on the scanning process. The effective time in which cyclododecane can be used as an opacifier for acquisition purposes was estimated. Nowadays, there are sublimation materials developed especially for 3D scanning on the market, which are listed as follows: Aesub (Scanningspray Vertriebs GmbH, Recklinghausen, Germany), Reflecon Type 1 (MR Chemie GmbH, Unna, Germany), and Attblime (Graichen Produktions- und Vertriebs GmbH, Bensheim, Germany) [14,15,16]. Currently, the only information about these materials is stated in the datasheets provided by their manufacturers, i.e., currently, there is a knowledge gap in the investigation and verification of their properties in terms of matting efficiency, thickness, or sublimation time.

The main goal of this work is a comparison of a wide range of commercially available sublimation scanning sprays. This work verified the effect of the number of applied coating layers on the 3D scanning results and coating thickness. This study also investigated the overall one-layer coating sublimation time. The work considered constant conditions for all experiments and coating deposition to maximize objectivity when comparing individual materials.

## 2. Materials and Methods

Scanning sprays used in this work are stated in Table 1. Currently available commercial sprays that were dedicated as sublimation scanning sprays were used for the purpose of this work. The abbreviations stated in Table 1 are used further in this work for a distinction of the sprays. All tested materials were delivered as aerosol spray cans. In the right column, sublimation times declared by the manufacturers of the scanning sprays are also mentioned. The sprays RefT50, AtAB6, AtAB2, and AtAB0 are cyclododecane-based, while the other sprays are based on other materials. The exact composition of the products is not specified by the manufacturers.

**Figure 1:**
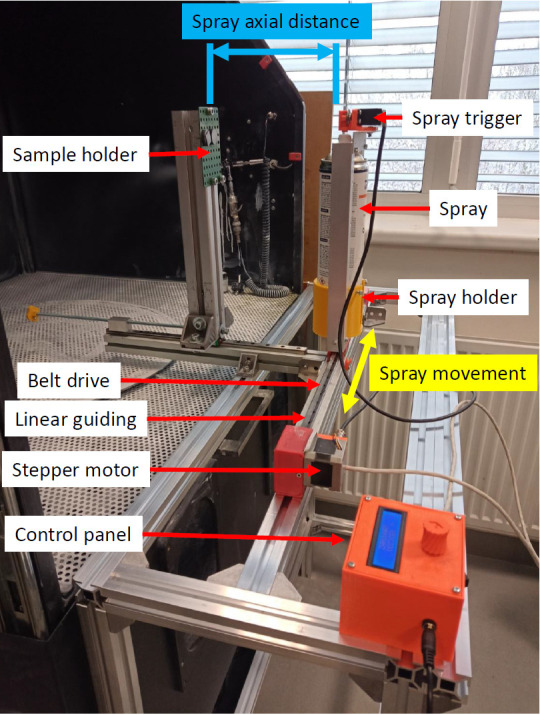
Coating deposition system.

An automated coating deposition system was used to ensure uniform conditions for coating application (see Figure 1).

Spray movement was ensured by a stepper motor with a belt drive and linear guiding. Coating deposition from the spray can was turned on using a servo motor with a trigger placed on the top of the spray holder (see Figure 1). The trigger intensity was set the same for all used scanning sprays. Spray movement speed, number of deposited coating layers, and triggering of the spray were driven by a microcontroller Arduino placed in the control panel. The spray movement speed was set at a constant 800 mm/s for all performed experiments. The spray axial distance was also set as constant, and all coatings were deposited at a distance of 175 mm from the spray nozzle. The spray movement speed and deposition distance were set experimentally to avoid over-spraying after one spray pass. Coating deposition was conducted perpendicularly to the coated surfaces, and the spray axis was directed at the center of the sample. Four samples were created for each measurement. All experiments were performed under constant ambient conditions. The ambient temperature for all experiments was 21 ± 1 °C. All experiments were performed 2 min after the coating deposition to eliminate the effect of the ambient environment and the time-dependent behavior of the coatings.

### 2.1. Coated Objects

A highly reflective Si-wafer (15 × 15 × 0.5 mm) was used for the surface coverage analysis, the analysis of the matting efficiency, and the measurement of the sublimation time. The Si-wafer was chosen for its excellent flatness (<400 nm) and low roughness (Ra ≈ 1 nm). The flatness and roughness of the Si-wafer were measured by the stylus profiler Bruker DektakXT (Bruker, Billerica, MA, USA), with a stylus radius of 12.5 μm, a stylus force of 3 mg, and a scan duration of 160 s. The surface of the Si-wafer is impossible to scan without a matte coating, which was necessary for matting efficiency analysis. Excellent flatness and low roughness were necessary to determine the effect of matte coating on the 3D scan quality. 

A white ceramic gauge block with a nominal dimension of 30 mm (30 × 35 × 9 mm) was used for the measurement of the coating thickness. It was used because its surface is possible to scan without matte coating and for its precise geometry.

Objects used for the coating deposition were not pretreated before the matting process. Wiping with the cloth was performed before testing to remove the dust and other impurities from the test surface. This corresponds to a common workflow during the 3D scanning process.

### 2.2. 3D Scanning

Atos III Triple Scan (Carl Zeiss GOM Metrology GmbH, Braunschweig, Germany) was used for 3D scanning. The 3D scanner was used for the verification of the effect of the coating on the 3D scan quality and the coating thickness measurement. The process of 3D scanning was automated using an assembly with the lift and rotation table to ensure uniform conditions during measurements, and each sample was scanned in one shot. Scanning details are mentioned in Table 2. A 3D scanning setup differed for the Si-wafer and ceramic gauge block measurement.

#### 2.2.1. 3D Scanning of the Si-Wafer

The Si-wafer was scanned by a measuring volume MV60, with perpendicular scanning orientation between the scanner and wafer. MV60 offered the best possible measuring point distance. Perpendicular orientation offered the worst scanning scenario when the light was reflected directly on the cameras of the scanner. This measurement was made to investigate the effect of matte coating on the 3D scan quality and its ability to improve the 3D scanning of the highly reflective surface. The analysis of the 3D scans was performed in the software GOM Inspect (Carl Zeiss GOM Metrology GmbH, Braunschweig, Germany). The coating ability to improve 3D scanning was evaluated by measuring the area captured by the scanner and the number of holes in the scanned mesh. The size of the evaluated area was 12 × 12 mm. The 3D scan quality was evaluated by the standard deviation (*Sigma*) of the captured point cloud against the fitted plane. The fitted plane was created using the Gaussian Best-fit method, with a selection of points in the range of 3σ.

#### 2.2.2. 3D Scanning of the Ceramic Gauge Block

**Figure 2:**
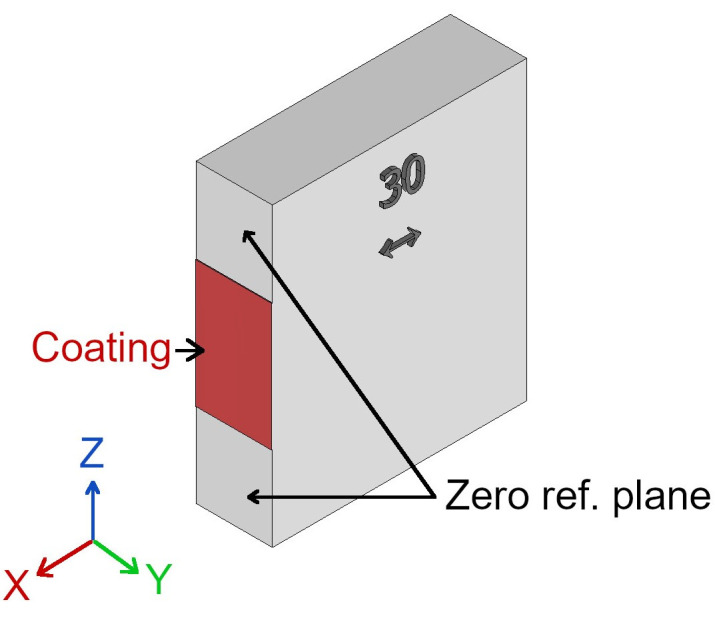
Scheme of the coating deposition on the gauge block.

The gauge block was scanned by measuring volume MV170 due to its larger dimensions. In this case, the scanning orientation was set as 45° to eliminate problematic direct reflections during 3D scanning. This measurement was conducted to investigate the coating thickness. The use of 3D scanner for the coating thickness measurement was motivated by the following statements: Firstly, 3D scanning results can show the effect of the coating on the 3D scanning accuracy, i.e., 3D scanning results can show if the coating is detectable by the scanner. Secondly, the 3D scanner can capture the entire coated surface in one shot. It was necessary to eliminate the time-dependent behavior of deposited coatings. The gauge block was coated in the middle of the surface, defining its nominal dimension (see Figure 2). The width of the coated area was 15 mm. The rest of the surface remained uncoated, which defined a zero reference plane. This was achieved using a clamping mask during the coating deposition.

The coating thickness was measured in the software GOM Inspect. The distance between the coated area and the zero reference plane was measured using the Projected Point Distance function. The measurement was conducted for 9 measuring points. The first measuring point was in the center of the coated area. The rest of the measuring points were offset from the center point by ±1.5 mm in the *Y* direction and by ±5 mm in the *Z* direction.

### 2.3. Coverage of the Si-Wafer by the Coating

**Figure 3:**
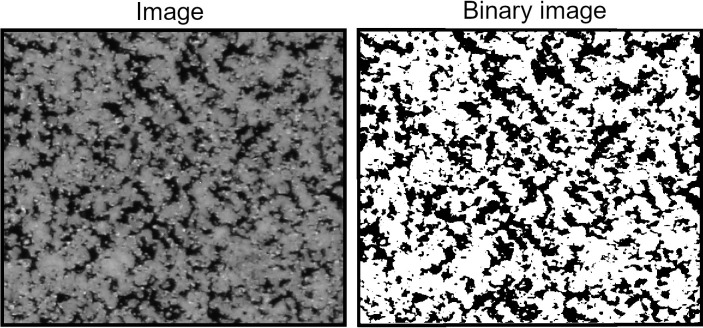
Conversion of the captured image into the binary image.

The analysis of the coverage of the Si-wafer by the coating was performed to determine the amount of deposited coating material. Coatings were captured by an optical microscope Olympus SZX7 (Olympus, Tokyo, Japan). Magnification was set as 1. The percentage overlap of the wafer surface by the coating was measured in the software ImageJ, Version 1.53t (NIH, Bethesda, MD, USA and LOCI, University of Wisconsin-Madison, Madison, WI, USA) using the Measure function. For this analysis, captured images were converted into a binary image by thresholding (see Figure 3). White spots in Figure 3 represent the coating. The thresholding was also performed in the ImageJ software, Version 1.53t, where the threshold was set using the Yen algorithm [17].

### 2.4. Sublimation Time Measurement

The sublimation time measurement was performed in the closed box (180 × 120 × 130 mm), which eliminated the effect of the ambient environment. Sublimation time was measured based on the images taken by the camera Imaging Source DMK 23U618 (The Imaging Source, LLC, Charlotte, NC, USA) with the lens Computar T0812FICS-3 (CBC Group, Tokyo, Japan). Samples were photographed every 60 s, and the sublimation time was computed from the time stamps of the taken photos. Measurements were automated using Python script and microcontroller Arduino. The sublimation time represents the moment when the coating completely disappeared from the surface of the Si-wafer after its deposition.

## 3. Results and Discussion

### 3.1. Coverage of the Si-Wafer by the Coating

**Figure 4:**
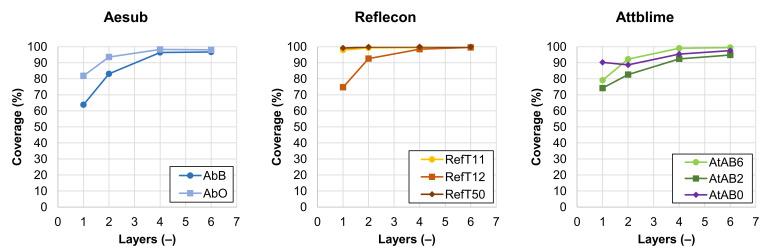
Dependence of the coverage of the Si-wafer by the coating on the number of applied coating layers.

The graphs in Figure 4 show the average coverage of the Si-wafer depending on the number of applied coating layers. The results show that the coverage ability differed between individual sprays. It was the most obvious in the case of the one-layer coating. The highest coverage ability was provided by sprays RefT11 and RefT50, which reached the coverages of 97.9 ± 0.8% and 99.2 ± 0.2% with only the one-layer coating. On the contrary, the lowest average coverage with the one-layer coating was provided by the spray AbB (63.8 ± 2.7%).

It was assumed that the coverage ability could be coupled not only with the composition of the sprayed material but also with the volumetric flow rate of the material in the created spray. The spray cans were weighted by the digital balance Jadever JKH-500 (Jadever Scale Co., Ltd., New Taipei City, Taiwan) before and after the coating deposition to track the loss of the material. The weight losses of the material in the spray cans after the one spray crossing over the coated object (i.e., after deposition of the one-layer coating) are stated in Table 3. It is obvious that the spray AbB provided the lowest weight loss, which was probably reflected in its lower coverage ability with the one-layer coating. It was also observed that the sprays RefT12, AtAB6, AtAB2, and AtAB0, despite their relatively high weight losses, provided less coverage ability with their one-layer coatings. These one-layer coatings were visually less homogeneous and contained more imperfections compared to the coatings deposited by the sprays RefT11, RefT50, and AbO.

**Figure 5:**
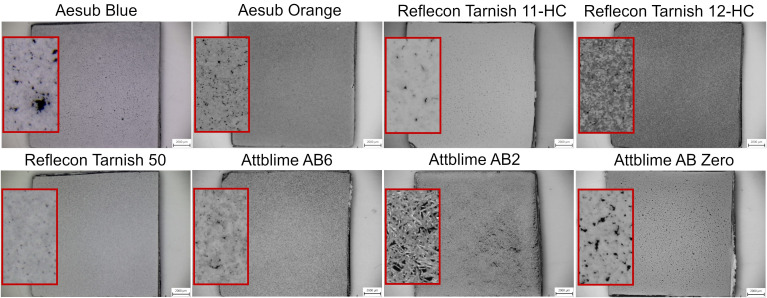
Images of the 4-layer coatings.

The results in Figure 4 also show that the four-layer coating led to coverage greater than 95% for almost all used scanning sprays. From Figure 4, it is also clear that the sprays AbB, AtAB2, and AtAB0 did not reach such coverage at higher numbers of layers compared to other tested materials. This was coupled with imperfections in the coatings. In this case, larger holes and cracks were more frequently present in the morphology of the coatings (see Figure 5). A hypothesis explaining the crack formation in these coatings was set. It was assumed that the cracks were formed by solvent that is present in the sprays. If there is a larger amount of the solvent delivered on the coated surface, it disrupts the structure of the coating that is already present on the surface, leading to the creation of the coating imperfections. Figure 5 shows images of the four-layer coatings deposited on the Si-wafer. Details of the coatings are shown in the red squares. It is obvious that the coating morphology differed for each spray.

### 3.2. 3D Scanning

**Figure 6:**
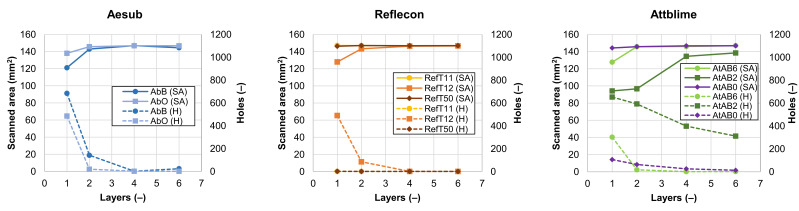
The dependence of the scanned area and number of holes in the scanned mesh on the number of applied coating layers.

**Figure 7:**
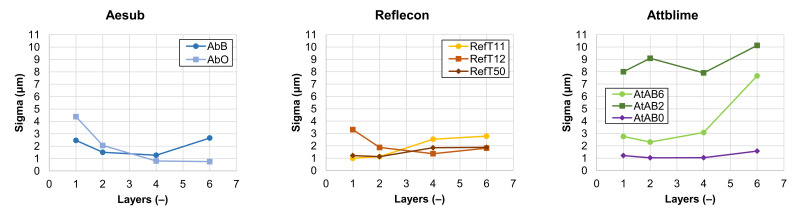
The dependency of the scanned point cloud Sigma on the number of applied coating layers.

The coating quality was coupled with the results of 3D scanning. Lack of coverage led to incomplete 3D scans with low quality. The results of 3D scanning are summarized in the graphs shown in Figure 6 and Figure 7. Figure 6 shows a dependency of the scanned area (SA) and the number of holes (H) in the captured mesh on the number of applied coating layers. The maximal scanned area was 144 mm^2^, which was defined by the selected control area 12 × 12 mm.

The results of the 3D scan quality analysis are shown in Figure 7. Here, the standard deviation (*Sigma*) of the scanned point cloud in dependency on the number of applied coating layers is stated. The graphs show the average values.

From the 3D scanning results, it is clear that most of the sprays showed similar trends in their behavior. The increasing number of applied coating layers increased the coverage of the wafer (see Figure 4), leading to the scanning of the whole control area with a reduced number of holes (see Figure 6). The results in Figure 7 also have similar trends for most of the used sprays. The *Sigma* trends generally consist of the downtrend, the minimum, and the uptrend. An explanation of each part of the *Sigma* trend is provided by the following hypothesis. The decreasing trend of the *Sigma* was coupled with the elimination of the problematic reflections of the light from the mirror-like surface of the Si-wafer by the coating. The increasing trend of the *Sigma* value was then coupled with a microstructure of the coating, which can be more noticeable with more coating layers. The minimum of the graph indicates the coating with the best parameters from the 3D scan quality point of view. Although the results had similar trends for most of the used sprays, the numerical results differed.

Coatings applied by the sprays RefT11 and RefT50 enabled the scanning of the whole surface without holes starting with the one-layer coating. The trends in Figure 6 are for the sprays RefT11 and RefT50 and are more or less the same. The results in Figure 7 show that coatings deposited from these sprays can be sensitive to over-spraying (see the increasing *Sigma* value). The sprays AtAB6 and AbB were also sensitive to over-spraying (see Figure 7). The spray AtAB6 provided the best scan quality with the two-layer coating, where the *Sigma* value was at its minimum. More coating layers resulted in considerable degradation of the 3D scan quality influenced by the rough microstructure of the coating. On the other hand, more layers eliminated the holes in the mesh. AbB had the best scanning results with the four-layer coating. In this case, more coating layers (over-spraying) led to extensive cracks in the coating morphology, resulting in a reduced scanned area and increased number of holes in the mesh (see Figure 6). It also negatively affected the 3D scan quality (see the increasing *Sigma* value in Figure 7). The four-layer coating also worked well in the case of the spray RefT12. The control area was captured completely, holes were eliminated, and the *Sigma* value was at its minimum. The spray AbO provided the best scanning results with a higher number of coating layers. The six-layer AbO coating provided the scans with the best quality of all tested sprays. Three-dimensional scans with low *Sigma* values were also offered by the spray AtAB0. However, AtAB0 coatings contained imperfections that negatively affected the captured scans, which contained holes. The worst scanning results were from the spray AtAB2. Even the six-layer coating was not sufficient to capture the control area completely. Three-dimensional scans contained a large number of holes, and the *Sigma* values were the highest of all used sprays. It is clear from Figure 5 that the AtAB2 coating morphology appeared different compared to the other materials. In this case, the coatings consisted of small needle-like clusters, which probably had a lower ability to eliminate the problematic reflections during 3D scanning. It was assumed that such a behavior was coupled with the composition of the scanning spray AtAB2.

### 3.3. Coating Thickness

**Figure 8:**
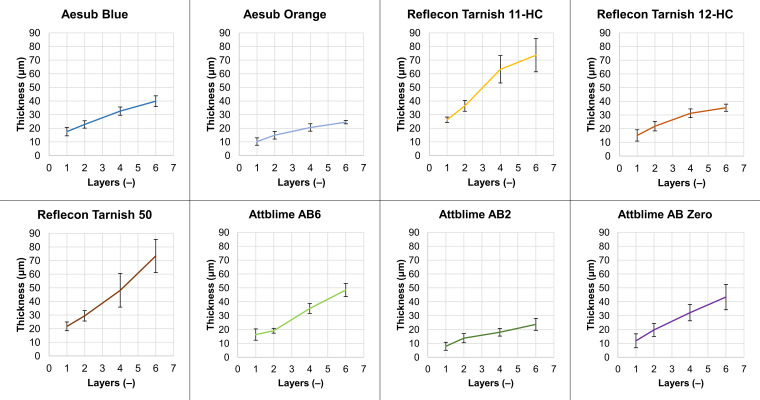
The dependence of the coating thickness on the number of applied coating layers.

The results of the coating thickness measurement are shown in Figure 8. The graphs show the average values and standard deviations (black lines). Figure 8 shows that all used sprays created coatings with considerable thicknesses detectable by the scanner. This suggests that all tested scanning sprays must be used carefully because thicknesses in the range of tens of micrometers can influence the accuracy of 3D scanning. The coating thickness in the range of tens of micrometers was also typical in the studies of Paloušek and Burde [9,11], where the non-sublimation scanning sprays were investigated. It is worth saying that when the maximum possible scanning accuracy is required, TiO_2_-based coatings are still the most suitable. According to the work of Franke [10], TiO_2_-based coatings can be deposited with thicknesses lower than 1 µm.

The thinnest coatings in this study were provided by the sprays AbO and AtAB2, which, even in the case of the six-layer coating, did not reach a value of 30 µm. The highest coating thicknesses were observed for the sprays RefT11 and RefT50, which were coupled with their high coverage ability.

The results of the 3D scanning and thickness measurement are summarized in Table 4. This table compares the sprays with each other and summarizes their matting ability. Stated data were chosen based on the minimal number of holes in the captured meshes. The average values of the *Coverage*, *Sigma,* and *Thickness* are stated with the standard deviations. The *Holes* values in Table 4 are stated as the average values. Table 4 shows that only the sprays AbO, RefT11, RefT12, RefT50, and AtAB6 were able to eliminate holes in the captured mesh. In the case of the spray AbB, the holes were almost eliminated with the four-layer coating. Obtained data suggest that the best results were provided with the spray AbO, which offered the best ratio of 3D scan quality and coating thickness. However, its lower coverage ability can be limiting, because the six-layer coating was necessary to obtain such results.

**Figure 9:**
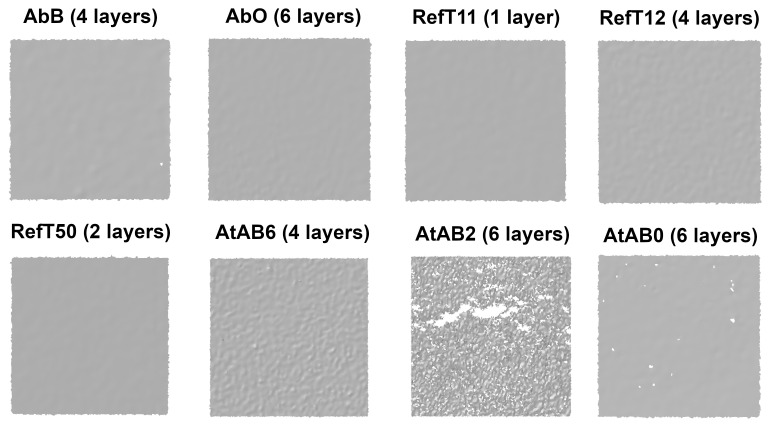
Three-dimensional scans of the coated Si-wafer.

Three-dimensional scans of the Si-wafer coated by the coatings with the number of layers according to Table 4 are shown in Figure 9. The gray areas represent the scanned mesh. In Figure 9, is obvious that higher *Sigma* values of the scans were obtained by scanning the AtAB6 and AtAB2 coatings. In this case, the mesh appeared uneven.

### 3.4. Sublimation Time

**Figure 10:**
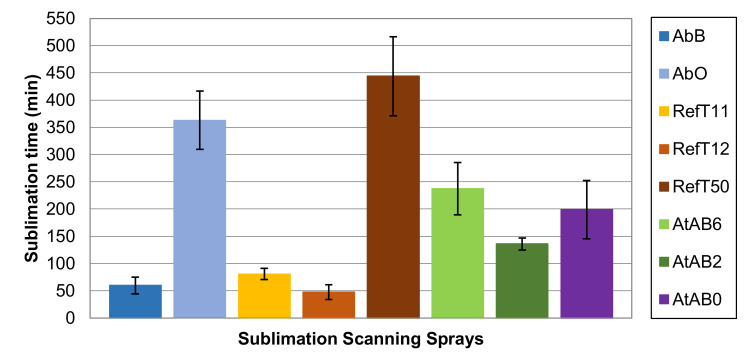
The sublimation time of the 1-layer coatings.

The time of complete disappearance for the one-layer coating was measured in this work. The results are shown in Figure 10. The bar plots show the average values and standard deviations (black lines).

Figure 10 shows that the sublimation times differed remarkably between tested scanning sprays. This was assumed from the values declared by the manufacturers in Table 1. However, there are differences when comparing measured data with those declared by manufacturers. These can be due to different conditions during measurements made by manufacturers. The number of applied layers (thickness of deposited coating), the coated object, the ambient environment, etc., may differ. 

**Figure 11:**
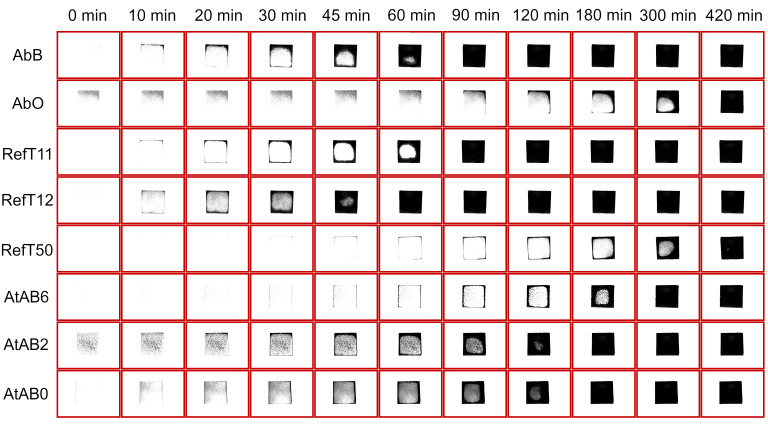
Sublimation process of the 1-layer coatings.

According to the results obtained in this study, tested scanning sprays can be generally divided into three groups according to their sublimation time. The first group of sprays consists of AbB, RefT11, and RefT12 and can be classified as fast-sublimating sprays. In this case, the sublimation time was less than 90 min. Specifically, AbB sublimated after 60 ± 16 min, RefT11 after 81 ± 10 min, and RefT12 after 47 ± 14 min. The second group of sprays consists of AtAB6, AtAB2, and AtAB0 and can be classified as medium-time sublimation sprays. The results show that these sprays provided sublimating times that were approximately in the middle of all used sprays. Sublimation times were 237 ± 48 min for AtAB6, 136 ± 11 min for AtAB2, and 199 ± 54 min for AtAB0. The last group of the sublimation scanning sprays consists of AbO and RefT50. A long sublimation time was typical for these sprays. AbO sublimated approximately 363 ± 54 min, while RefT50 sublimated 444 ± 73 min. Figure 11 shows images of the coatings taken during the sublimation time measurement. The sublimation process of the coating from the Si-wafer for AbB and AbO materials can also be seen in Videos S1 and S2 (from Supplementary Material), respectively. The dark areas represent the surface of the Si-wafer. The sublimation process was the same for all used sprays. The sublimation started on the edges of the Si-wafer and continued to the center of its surface. The only difference was in the sublimation rate. It was also apparent that the sublimation ended in the areas where the largest amount of the coating material was applied. This was generally in the areas where the center of the spray went through the surface of the Si-wafer. However, the trend of the sublimation process also showed that sublimation is influenced by the exposition of the coating edges to the ambient environment.

The results in Figure 11 suggest that the time-dependent behavior of the coatings must be considered when a 3D scanning strategy is planned. Short-time sublimating sprays seem to be suitable for fast scanning. The results showed that one-layer coatings started to disappear from the edges of the wafer after approximately 10 min, and the surface of the Si-wafer was, after some time, coated incompletely. If such a sublimation rate is too high for 3D scanning, the application of more coating layers or re-spraying can solve this problem. Coatings with longer sublimation times lasted longer for the entire surface of the Si-wafer. This is suitable for scanning complex parts with no need to re-spray the surface during scanning. 

**Figure 12:**
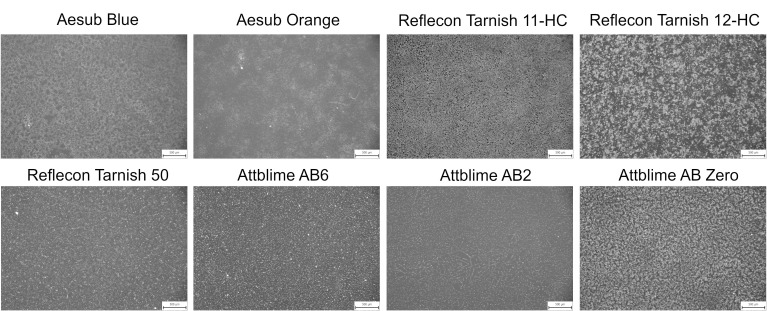
Detailed images of the Si-wafer surface after the coating sublimation.

The obtained results showed that all used materials vanished from the surface of the Si-wafer completely. However, it was observed that all coatings left visual traces after their disappearance. The traces on the surface of the Si-wafer were captured by the optical microscope Olympus SZX7 (Olympus), with a magnification of 5.6 (see Figure 12). The bright spots in Figure 12 represent traces left by the vanished coating, and the scales shown in the corners of the images have a value of 500 µm. Traces were present in the areas where the coating clusters were present before their sublimation. A hypothesis for further verification was set, stating that the traces were remains of the components contained in the sprays (e.g., solvents) that left visual marks after the coating disappearance. It is worth saying that these traces were apparent due to the smooth mirror-like surface of the Si-wafer and were easy to remove by wiping with a cloth.

## 4. Conclusions

This study focused on the comparison of eight sublimation scanning sprays in terms of their effect on the 3D scanning results, coating thickness, and sublimation time. All coatings were applied by the automated system, and all experiments were performed under constant ambient conditions, two minutes after their deposition. The ability of the sublimation coating to improve 3D scanning was investigated on the highly reflective surface of the Si-wafer. The coated surface was reconstructed by the structured light 3D scanner. This study proved that all used materials were able to improve the optical properties of the Si-wafer surface. The results also showed that sublimation scanning sprays can be a suitable alternative for scanning problematical surfaces when the cleaning process must be eliminated or excluded from the scanning process. However, the differences between some sprays were significant. The general results of this study are as follows:

- All tested sprays created coatings with a thickness in the order of tens of micrometers. This means that they are suitable for 3D scanning when a lower accuracy is required.
- All tested materials vanished from the surface of the Si-wafer completely. However, all coatings left traces on the smooth mirror-like surface of the Si-wafer after their sublimation.
- Tested sprays were divided into three categories according to their sublimation time. Short-time sublimation sprays (AbB, RefT11, and RefT12): sublimation time did not exceed 90 min. Middle-time sublimation sprays (AtAB6, AtAB2, and AtAB0): sublimation times were in the range of 130–300 min. Long-time sublimation sprays (AbO and RefT50): sublimation times were in the range of 300–500 min. The values correspond to the one-layer coatings.
- The best 3D scanning results were obtained with the six-layer coating applied by the spray AbO. The standard deviation of the point cloud (*Sigma*) of 0.75 µm was the lowest, the Si-wafer surface was reconstructed completely without holes, and the coating thickness was, in the case of six-layer coating, around 24 µm.
- The lowest ability to improve the highly reflexive surface for 3D scanning was provided by the spray AtAB2. Although this spray created one of the thinnest coatings, its coatings led to the scanning results with the lowest quality. The scans contained holes, and the *Sigma* values were around 9 µm.

The future direction for this research topic can be seen in the investigation of the time-dependent behavior of the sublimation coatings and its effect on the 3D scanning results. Another direction can be the investigation of how the ambient environment and the type of coated object affect deposited coatings, their sublimation time, and the associated 3D scanning results. There is also a space for investigating sublimation scanning liquids that can be applied by spray guns. Spray gun can provide more stable deposition conditions and may allow the optimization of the coating deposition process for specific requirements of the resulting coating.

## Figures and Tables

**Table 1:** List of used sublimation scanning sprays.

Manufacturer	Spray Type	Abbreviation	Sublimation Time (min)
Scanningspray Vertriebs GmbH	Aesub Blue	AbB	240
	Aesub Orange	AbO	720–1440
MR Chemie GmbH	Reflecon Tarnish 11-HC	RefT11	60–180
	Reflecon Tarnish 12-HC	RefT12	60–120
	Reflecon Tarnish 50	RefT50	300–480
Graichen Produktions- und Vertriebs GmbH	Attblime AB6	AtAB6	360–600
	Attblime AB2	AtAB2	120–240
	Attblime AB Zero	AtAB0	60–120

**Table 2:** The 3D scanning details.

General Parameters		
Sensor	ATOS III Rev. 02	
Camera resolution	2 × 8 Mpx	
Projector light	Blue	
Projection	Structured light with phase shift	
**3D scanning setup**		
Scanned object	**Si-wafer**	**Gauge block**
Measuring volume	MV60	MV170
Camera position	SO	SO
Measuring volume dimensions	(60 × 45 × 35) mm	(170 × 130 × 130) mm
Measuring point distance	0.017 mm	0.055 mm
Measuring distance	490 mm	490 mm
Exposure time	50 ms	35 and 70 ms
Certificate VDI/VDE 2634 Part 3		
Probing error form (sigma)	0.001 mm	0.001 mm
Probing error (size)	0.002 mm	−0.005 mm
Sphere spacing error	−0.001 mm	0.002 mm
Automated scanning	Lift and rotation table	
Scanning orientation	0°	45°
Polygonization and post-processing	Standard	

**Table 3:** The loss of material in the spray can after the deposition process of the one-layer coating.

Spray	Loss of Material (g)
AbB	0.77 ± 0.16
AbO	0.98 ± 0.10
RefT11	1.54 ± 0.21
RefT12	1.45 ± 0.17
RefT50	1.41 ± 0.17
AtAB6	1.32 ± 0.24
AtAB2	1.39 ± 0.12
AtAB0	1.72 ± 0.23

**Table 4:** The best coating parameters for the 3D scanning of the highly reflexive Si-wafer.

Spray	Layers (−)	Coverage (%)	Holes (−)	Sigma (µm)	Thickness (µm)
AbB	4	96.4 ± 1.2	1	1.27 ± 0.18	32.6 ± 3.0
AbO	6	98.0 ± 0.6	0	0.75 ± 0.13	24.5 ± 1.2
RefT11	1	97.9 ± 0.8	0	0.99 ± 0.24	26.2 ± 2.0
RefT12	4	98.4 ± 0.8	0	1.36 ± 0.10	31.2 ± 3.2
RefT50	2	99.8 ± 0.1	0	1.13 ± 0.20	29.4 ± 3.8
AtAB6	4	99.0 ± 0.4	0	3.08 ± 0.71	35.2 ± 3.5
AtAB2	6	94.8 ± 3.5	311	10.13 ± 1.43	23.7 ± 4.3
AtAB0	6	97.6 ± 0.6	13	1.58 ± 0.31	43.4 ± 9.2

## Data Availability

Not applicable.

## Supplementary Materials

The following supporting information can be downloaded at: https://www.mdpi.com/article/10.3390/ma16186165/s1, Video S1: Sublimation process from the Si-wafer for AbB; Video S2: Sublimation process from the Si-wafer for AbO.

## References

[1] Sansoni G., Trebeschi M., Docchio F. (2009). State-of-the-art and applications of 3D imaging sensors in industry, cultural heritage, medicine, and criminal investigation. Sensors.

[2] Pereira J.R.M., De Lima E Silva Penz I., Da Silva F.P. (2019). Effects of different coating materials on three-dimensional optical scanning accuracy. Adv. Mech. Eng..

[3] Wang Y., Feng H.-Y. (2014). Modeling outlier formation in scanning reflective surfaces using a laser stripe scanner. Meas. J. Int. Meas. Confed..

[4] Chen T., Lensch H.P.A., Fuchs C., Seidel H.P. Polarization and phase-shifting for 3D scanning of translucent objects. Proceedings of the 2007 IEEE Conference on Computer Vision and Pattern Recognition.

[5] Zaimović-Uzunović N., Lemeš S. Influences of Surface Parameters on Laser 3D Scanning. Proceedings of the IMEKO Conference Proceedings: International Symposium on Measurement and Quality Control 2010.

[6] Ihrke I., Kutulakos K.N., Lensch H.P.A., Magnor M., Heidrich W. (2010). Transparent and specular object reconstruction. Comput. Graph. Forum.

[7] Rukosuyev M.V., Barannyk O., Oshkai P., Jun M.B.G. (2016). Design and application of nanoparticle coating system with decoupled spray generation and deposition control. J. Coat. Technol. Res..

[8] Maeng H.-Y., Lee M. (2016). A study on the exploration of electrostatic powder coating materials suitable for 3D scanning. MATEC Web Conf..

[9] Paloušek D., Omasta M., Koutný D., Bednář J., Koutecký T., Dokoupil F. (2015). Effect of matte coating on 3D optical measurement accuracy. Opt. Mater..

[10] Franke J., Koutecký T., Malý M., Kalina M., Koutný D. (2022). Study of process parameters of the atomizer-based spray gun for the application of a temporary matte coating for 3D scanning purposes. Mater. Chem. Phys..

[11] Burde A.V., Dudea D., Cuc S., Moldovan M., Campian R.S. (2016). Three—Dimensional evaluations of the coating thickness of two optical conditioning scanning sprays. Mater. Plast..

[12] Yang Y., Chen S., Wang L., He J., Wang S.M., Sun L., Shao C. Influence of coating spray on surface measurement using 3D optical scanning systems. Proceedings of the ASME 2019 14th International Manufacturing Science and Engineering Conference, MSEC 2019.

[13] Díaz-Marín C., Aura-Castro E., Sánchez-Belenguer C., Vendrell-Vidal E. (2016). Cyclododecane as opacifier for digitalization of archaeological glass. J. Cult. Herit..

[14] Scanningspray Vertriebs GmbH Aesub—State of the Art Scanningspray. https://aesub.com/en/.

[15] MR Chemie GmbH Reflecon—3D Scanningspray. https://www.mr-chemie.de/en/reflecon-3d-scanningspray/.

[16] Graichen Produktions- Und Vertriebs GmbH Attblime. https://attblime.com/?lang=en.

[17] ImageJ Auto Threshold. https://imagej.net/plugins/auto-threshold.

